# Commercial Bovine Proteoglycan Is Highly Arthritogenic and Can Be Used as an Alternative Antigen Source for PGIA Model

**DOI:** 10.1155/2014/148594

**Published:** 2014-05-27

**Authors:** Larissa Lumi Watanabe Ishikawa, Priscila Maria Colavite, Larissa Camargo da Rosa, Bianca Balbino, Thais Graziela Donegá França, Sofia Fernanda Gonçalves Zorzella-Pezavento, Fernanda Chiuso-Minicucci, Alexandrina Sartori

**Affiliations:** Department of Microbiology and Immunology, Biosciences Institute, São Paulo State University (UNESP), 18618-000 Botucatu, SP, Brazil

## Abstract

Rheumatoid arthritis (RA) is the most common systemic autoimmune disease. It affects mainly the joints, causing synovitis, cartilage destruction, and bone erosion. Many experimental models are used to study the mechanisms involved in immunopathogenesis and new therapies for this disease. Proteoglycan-induced arthritis (PGIA) is a widely used model based on the cross-reactivity of injected foreign (usually human) PG and mice self-PG. Considering the complexity of the extraction and purification of human PG, in this study we evaluated the arthritogenicity of bovine PG that is commercially available. Bovine PG was highly arthritogenic, triggering 100% incidence of arthritis in female BALB/c retired breeder mice. Animals immunized with bovine PG presented clinical symptoms and histopathological features similar to human RA and other experimental models. Moreover, bovine PG immunization determined higher levels of proinflammatory and anti-inflammatory cytokines in arthritic mice compared to healthy ones. As expected, only the arthritic group produced IgG1 and IgG2a antibodies against PG. Thus, commercial bovine PG can be used as an alternative antigenic source to PGIA for the study of many RA aspects, including the immunopathogenesis of the disease and also the development of new therapies.

## 1. Introduction

Rheumatoid arthritis (RA) is a chronic inflammatory disease that affects around 0.3 to 1% of the world population, with lower prevalence in developing countries [1]. It is considered the most common systemic autoimmune disease that usually affects the small joints, especially fingers. It may also involve larger joints, including shoulders, elbows, knees, and ankles. The inflammatory process in the joint is characterized by synovitis, cartilage destruction, and bone erosion. There is still no consensus on the autoantigens involved in this disease. Currently, it is known that some autoantigens such as cartilage components, chaperone proteins, enzymes, nuclear proteins, and citrullinated proteins might be involved [2, 3]. Among several cell types found in the inflamed joint, CD4+ T-cells' subsets are considered the most important cells involved in synovitis and RA development [4]. Activated macrophages are also a very relevant source of inflammatory mediators, including proinflammatory cytokines [5]. TNF-*α* and IL-1, for example, promote the accumulation of inflammatory cells in the joints and the synthesis of other cytokines, chemokines, and matrix metalloproteinases [6]. Many cytokines, including IL-8, TNF-*α*, and IFN-*γ*, have been detected in synovial fluid. These cytokines, especially TNF-*α*, activate resident synovial cells that by producing proteolytic enzymes mediate the destruction of joint cartilage, ligaments, and tendons. Recently, the presence of IL-17 at the site of inflammation and its synergistic effect with TNF-*α*, exacerbating the inflammatory process, have been evidenced [7]. The participation of B cells in RA has been more investigated in recent years. The production of autoantibodies and cytokines, presentation of autoantigens to T cells, and ectopic lymphoid organogenesis are their possible roles [8]. Regulatory T cells have also been widely studied in both human and experimental arthritis because of their therapeutic and prophylactic potential [9].

There are several experimental models of arthritis being used to elucidate the mechanisms involved in the immunopathogenesis of RA. Also, the animal models are essential to study new therapy targets for this disease. Historically, the first experimental models of arthritis, which are called adjuvant-induced arthritis (AIA), were based on the inoculation of mycobacterial components suspended in mineral oil. Later, it was discovered that this model could be improved by using pristane, which is a purified component of mineral oil. The disease caused by pristane was more similar to human RA and this model has been widely used [10–12]. After that, there was an increased interest in experimental models based on the inoculation of cartilage components such as type II collagen and proteoglycan. These models presented clinical, immunological, histopathological, and genetic characteristics typical from human RA and, for that reason, they were considered the best models to study mechanisms and possible treatments for arthritis [13, 14]. In the 90s, the first arthritis transgenic models were described. By using immunogenetic tools, Keffer et al. [15] observed a spontaneous arthritis in mice overexpressing human TNF-*α* transgene. In this study, the animals developed a chronic inflammatory polyarthritis that evidenced the critical role of TNF-*α* in the immunopathogenesis of RA. Currently, collagen-induced arthritis (CIA) is a very reliable and reproducible experimental model that is being widely used for the study of all aspects of arthritis, including the immunopathogenesis of RA, the development of new drugs from natural extracts, the new molecular targets for treatment, and also gene therapy [16–19].

The experimental model chosen for this study was based on the immunization of BALB/c mice with proteoglycan (PG). Proteoglycan-induced arthritis (PGIA) was elegantly described by Glant et al. [13]. Briefly, the systemic autoimmune arthritis in this model is induced by intraperitoneal inoculation of BALB/c or C3H mice with PG isolated from various sources. Many genetic and immunological aspects of PGIA have already been studied in this model. For example, epitopes recognized by the arthritogenic T cells and the contribution of various cytokines such as IFN-*γ*, IL-4, and IL-12 were determined [20–22]. Although human cartilage is the preferable source of PG, its extraction and purification is a complex and laborious process that includes a variety of biochemical steps. Besides, ethical issues and rules involving the utilization of biological samples from human and animals contribute to complicate PG purification. In this scenario, we investigated the possible arthritogenicity of bovine PG in BALB/c mice. We considered that this evaluation could be very beneficial to researchers that are not able to purify human PG. Commercial availability of bovine PG could not only facilitate the experimental model implementation but also facilitate the comparison of results obtained by different laboratories.

## 2. Materials and Methods

### 2.1. Animals

Female BALB/c retired breeder (beyond the reproductive age) mice were removed from breeding colonies by the age of 8–11 months and purchased from CEMIB (Campinas, SP, Brazil). They were maintained in the Department of Microbiology and Immunology facility under controlled conditions of luminosity (12 h light/12 h dark) and temperature (22 ± 2°C). Mice were allocated in ventilated cages with sterile pine shavings and received sterile food and filtrated water* ad libitum*. The manipulation of the animals was in compliance with the local ethics committee (Protocol number 257-CEEA).

### 2.2. Arthritis Induction and Score Evaluation

As previously described [23], with minor modifications, a dose (100 *μ*L) containing 100 *μ*g of bovine proteoglycan extracted from nasal septum (Sigma Aldrich, St. Louis, MO, USA) and 1 mg of emulsified (micelle form) dodecyl dioctadecyl ammonium bromide (DDA) adjuvant (Sigma Aldrich, St. Louis, MO, USA) was intraperitoneally injected three times with 21-day interval for arthritis induction. After the third injection, arthritis score was daily evaluated until euthanasia (70 days after the first immunization). Arthritis severity was determined using a standard visual scoring system based on the degree of swelling and redness ranging from 0 to 4 for each paw. The following system was used: 0 = normal; 1 = mild swelling in the paw or one joint; 2 = moderate swelling and redness in the paw and one or more joints; 3 = pronounced swelling and redness in the paw, all joints, and ankle; 4 = severe swelling and redness of the entire paw and ankle and movement limitation. This classification resulted in a total score that ranged from 0 to 16 for each animal.

### 2.3. Histopathological Analysis

After euthanasia, mice paws were collected and fixed in 10% formalin phosphate buffer for at least seven days at room temperature. The samples were thoroughly demineralized in 10% Titriplex EDTA disodium salt (Merck Millipore, Darmstadt, Germany) for one to two months. The decalcified tissues were trimmed, dehydrated in graded ethanol, and embedded in paraffin. Serial sections (5 *μ*m) were cut and mounted on glass slides precoated with 0.1% poly-L-lysine (Sigma Aldrich, St. Louis, MO, USA). Histological assessment was carried out following routine hematoxylin and eosin (HE) staining. The images were acquired by a digital camera attached to the optical microscope (Nikon, Kurobanemuko, Otawara, Japan).

### 2.4. Immune Responses Evaluation

For cellular immune response, spleens were collected after euthanasia and the cells resuspended in RPMI medium containing gentamicin and fetal calf serum. The cells were stimulated with ConA (5 *μ*g/mL) and PG (50 *μ*g/mL). After 48 hours incubation at 37°C/5% CO_2_, the supernatants were collected for detection of IL-2, IL-6, TNF-*α*, IL-17, IFN-*γ*, IL-5, and IL-10. These cytokines were quantified using enzyme linked immunosorbent assay (ELISA), according to the manufacturer's instructions (BD Biosciences, San Jose, CA, USA, and RD Systems, Minneapolis, MN, USA). For humoral immune response, blood samples were collected by facial vein two days before each dose and seven days after the third dose of PG+DDA. 70 days after the first immunization, the blood was collected by cardiac puncture. The sera were obtained by blood centrifugation (6000 rpm for 15 minutes at 25°C). Briefly, Maxisorp plates (Nunc, Life Technologies, USA) were coated with 5 *μ*g/mL of bovine PG (Sigma Aldrich, St. Louis, MO, USA) and nonspecific protein binding was blocked with 0.1% bovine serum albumin in phosphate buffered saline. Subsequently, plates were incubated with serum samples diluted 1 : 1000. Biotinylated anti-mouse IgG1 and IgG2a antibodies (BD Biosciences, San Jose, CA, USA) were used to detect heterologous anti-PG antibodies. Plates were then incubated with streptavidin (RD Systems, Minneapolis, MN, USA) and revealed by adding H_2_O_2_ and orthophenylenediamine (Sigma Aldrich, St. Louis, MO, USA).

### 2.5. Statistical Analysis

Results were presented as mean ± standard deviation for parametric variables and the comparison among the groups was performed by *t*-test. For nonparametric variables, the results were presented as median and the comparison between the groups was performed by Mann-Whitney's test. Paired *t* -test was performed for antibody production. All data were analyzed using SigmaPlot software version 12.0 (Jandel Corporation, USA) and *P* < 0.05 was considered significant.

## 3. Results

### 3.1. Arthritis Incidence and Clinical Score

**Figure 1:**
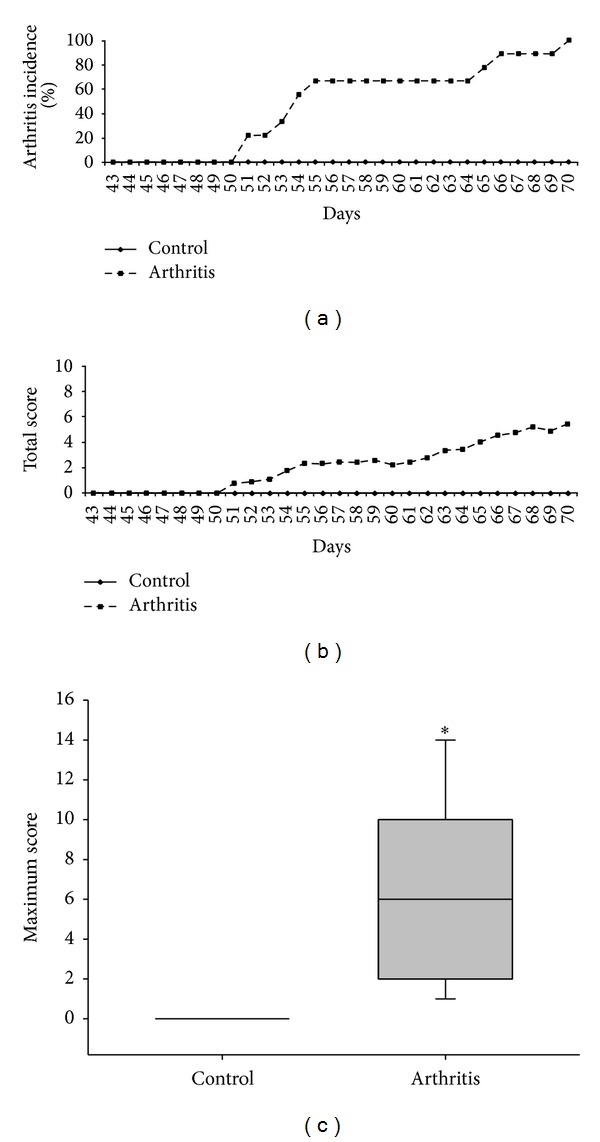
Arthritis incidence (a) total clinical score (b) and maximum clinical score (c) in mice with bovine proteoglycan-induced arthritis. Female BALB/c retired breeder mice were immunized with three doses of bovine PG associated with DDA adjuvant, 21-day interval. Clinical score was daily evaluated after the third immunization. *P < 0.05 compared to control.

As expected, animals from control group did not develop experimental arthritis. However, all animals immunized with three doses of bovine PG+DDA adjuvant developed the disease (Figure 1(a)). Arthritis onset was observed at day 51 and total clinical score increased in the arthritic group until day 70 (Figure 1(b)). Moreover, the median of the maximum score in the arthritic group was statistically significant in comparison to the healthy control group (Figure 1(c)).

### 3.2. Histopathological Analysis

**Figure 2:**
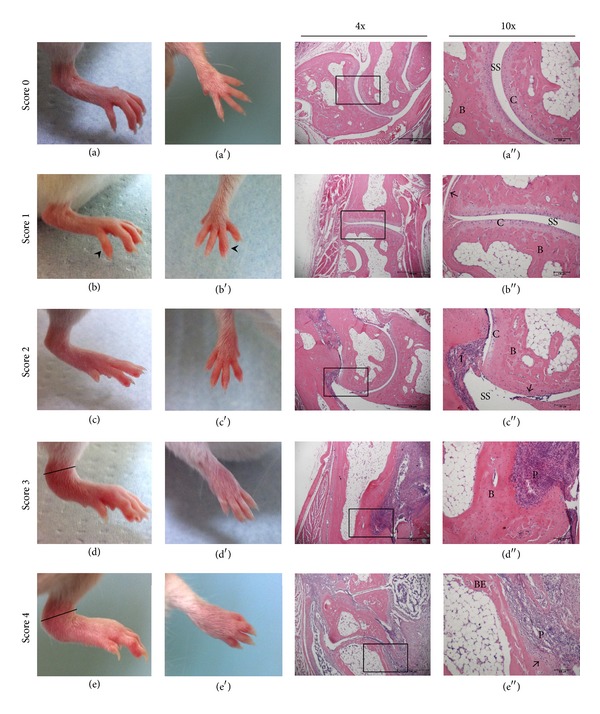
Representative clinical scores of hind paws (first column) and forepaws (second column) and histological sections of mice joints (third and fourth columns) with arthritis induced by bovine PG. Female BALB/c retired breeder mice paws were collected 70 days after the disease induction. The rectangles represent the regions highlighted in the fourth column. Head arrows indicate single joint inflammation; lines indicate ankle thickening; filled arrows indicate the synovial membrane. SS: synovial space; C: cartilage; B: bone; I: inflammatory infiltrate; P:* pannus*; BE: bone erosion.

Figure 2 shows the differences among the clinical scores observed in mice hind paws and forepaws during arthritis development. HE stained paw sections revealed important histological changes in the arthritic joints compared to the healthy ones. According to the scoring system, all animals from control group presented score 0 and there was no signal of inflammation in these animals (Figures 2(a) and 2(a′)). The joint structure was preserved and characterized by a well-defined synovial space, cartilage presence, thin synovial membrane, and compact bone (Figure 2(a′′)). Mice from arthritic group presented a variety of scores, ranging from 1 to 4 in each paw. Score 1 was characterized by only one inflamed joint (head arrows; Figures 2(b) and 2(b′)). No differences were observed in histological sections from paws with score 1; that is, all animals presented well preserved joint structures (Figure 2(b′′)). Score 2 was characterized by the presence of two or more affected joints in the paw (Figures 2(c) and 2(c′)). In this score, there was an inflammatory cell infiltrate and a slight thickening of the synovial membrane. However, it was still possible to observe the presence of the synovial space and well-preserved cartilage and bone tissue (Figure 2(c′′)). Score 3 was characterized by the inflammation of multiple joints including the ankle (lines; Figures 2(d) and 2(d′)). In this score, there was an inflammatory cells infiltrate that characterizes the initial* pannus* formation, which is the inflammatory tissue that invades the synovial space and promotes cartilage destruction and bone erosion (Figure 2(d′′)). However, bone tissue was still preserved in this score. Score 4 was characterized by accentuated erythema and edema throughout the foot and ankle, involving all joints, with consequent movement impairment (Figures 2(e) and 2(e′)). Inflammation and joint destruction were evident and were characterized by* pannus* formation, synovial membrane thickening, cartilage destruction, and bone erosion in paws with score 4 (Figure 2(e′′)).

### 3.3. Production of Cytokines

**Figure 3:**
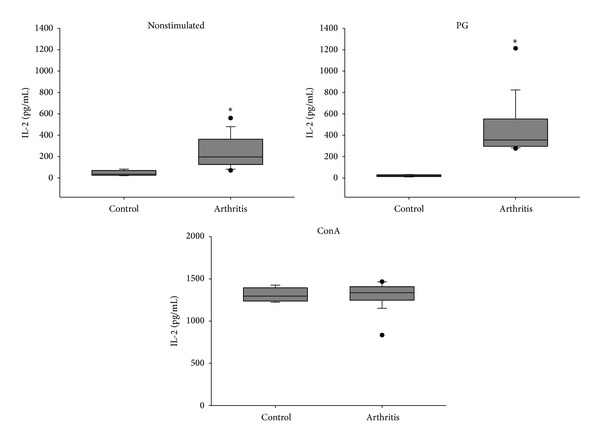
IL-2 production by spleen cells from BALB/c retired breeder mice with bovine proteoglycan-induced arthritis. Spleen cells were* in vitro* stimulated with PG and ConA and incubated for 48 hours. *P < 0.05 compared to the respective control.

**Figure 4:**
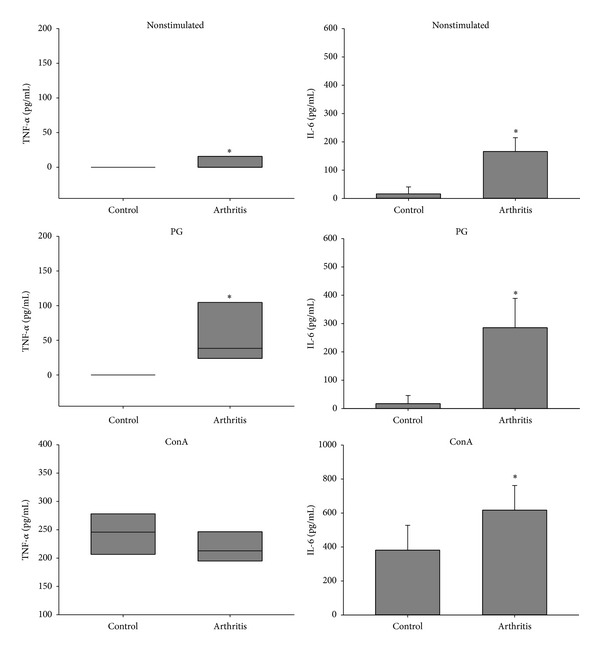
TNF-*α* and IL-6 production by spleen cells from BALB/c retired breeder mice with bovine proteoglycan-induced arthritis. Spleen cells were* in vitro* stimulated with PG and ConA and incubated for 48 hours. *P < 0.05 compared to the respective control.

**Figure 5:**
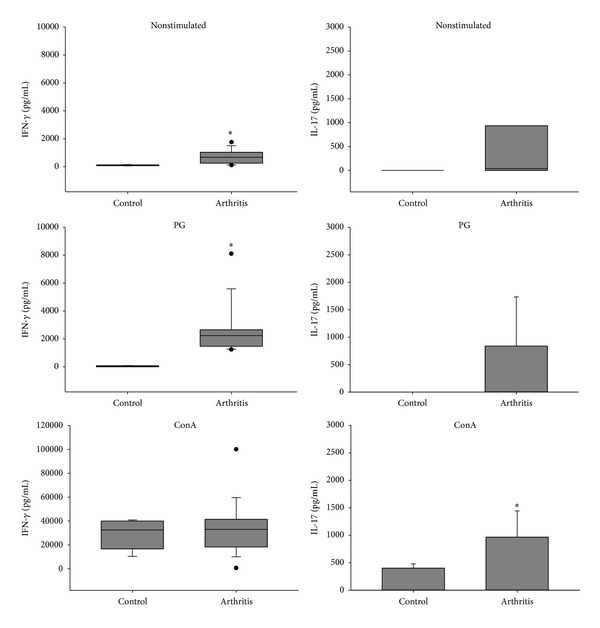
IFN-*γ* and IL-17 production by spleen cells from BALB/c retired breeder mice with bovine proteoglycan-induced arthritis. Spleen cells were* in vitro* stimulated with PG and ConA and incubated for 48 hours. *P < 0.05 compared to the respective control.

**Figure 6:**
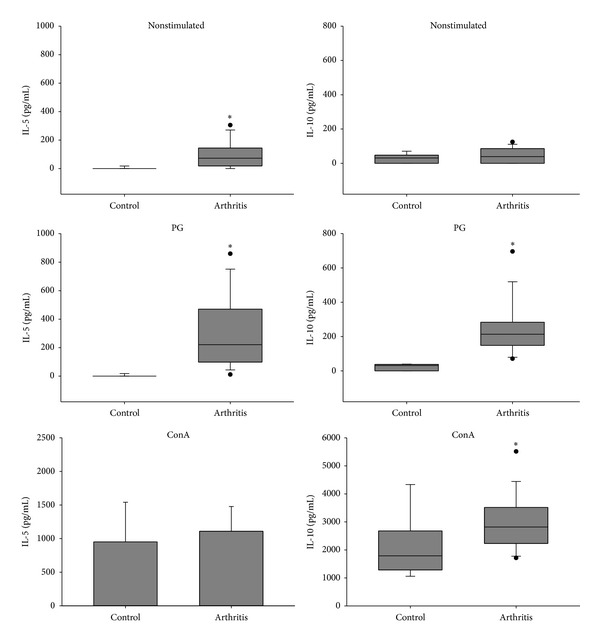
IL-5 and IL-10 production by spleen cells from BALB/c retired breeder mice with bovine proteoglycan-induced arthritis. Spleen cells were* in vitro* stimulated with PG and ConA and incubated for 48 hours. *P < 0.05 compared to the respective control.

Compared to the control group, spleen cells from arthritic mice produced significantly higher levels of IL-2 and the proinflammatory cytokines TNF-*α*, IL-6, IFN-*γ*, and IL-17 when restimulated* in vitro* with the specific antigen (Figures 3, 4, and 5). Interestingly, arthritic animals also produced significant levels of IL-5 and IL-10 anti-inflammatory cytokines in response to* in vitro* stimulation with PG (Figure 6). Results from nonstimulated cultures showed that there was spontaneous production of all cytokines in the arthritic animals, but not in the healthy ones (Figures 3, 4, 5, and 6). However, polyclonal stimulation of spleen cells with ConA triggered significant increase only in IL-6, IL-17, and IL-10 production by spleen cells from the arthritic group compared to the control one.

### 3.4. Production of Anti-PG Antibodies

**Figure 7:**
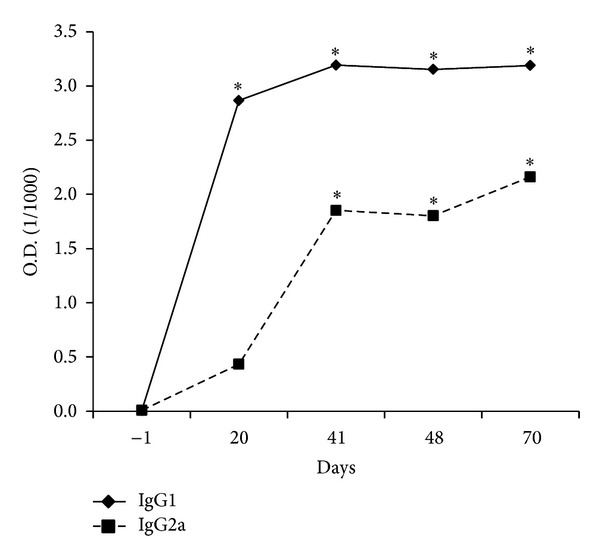
IgG1 and IgG2a serum levels from BALB/c retired breeder mice with bovine proteoglycan-induced arthritis. Blood samples were obtained two days before each PG+DDA immunization (days −1, 20, and 41), seven days after the third immunization (day 48), and after euthanasia (day 70). *P < 0.05 compared to day −1 of the same group.

The experimental arthritis induced by bovine PG determined production of both IgG1 and IgG2a anti-PG antibodies, with higher production of IgG1 (Figure 7). The levels of these specific antibodies increased significantly and progressively after the first immunization with PG+DDA (day 1). After reaching the maximum level around day 41, antibody production was maintained until euthanasia at day 70. As expected, control animals that were not immunized with PG+DDA did not produce specific antibodies against bovine PG (data not shown).

## 4. Discussion

There are several experimental models of rheumatoid arthritis that contribute to understand the mechanisms involved in this disease [24]. Experimental arthritis models induced by cartilage components have been extensively studied, primarily the induction of arthritis by collagen and proteoglycan (PGIA). Considering the clinical and histopathological characteristics of the disease, this model shares many similarities with human arthritis. The development of arthritis in PGIA is attributed to a cross-reaction against foreign PG and mice self-PG [25]. In this experimental model, the disease is usually triggered by injections of human PG associated with a strong adjuvant. The PG can be extracted from the cartilage of various origins, but human PG is considered the most arthritogenic one. In this context and considering that PG purification is a complex and laborious process, we determined the arthritogenicity of a commercial source of bovine PG. This evaluation was done by immunization of BALB/c retired breeders with three doses of bovine PG emulsified with DDA. In spite of the advanced age of these animals, no spontaneous arthritis was observed. According to Besenyei et al. [26], approximately 0.5 to 1.0% of retired breeder BALB/c mice can develop the disease spontaneously.

Immunization with the commercial bovine PG was very effective to induce arthritis. A 100% incidence was observed in the majority of the experiments as has been described with human PG [13, 23]. In terms of clinical disease, we observed slightly lower scores than the ones described for human PG. However, this finding was equally described by other authors that employed bovine PG [23, 27]. In spite of this, the histopathological analysis revealed the presence of very typical arthritic histological alterations as inflammatory infiltrates, synovial membrane thickening,* pannus* formation, cartilage destruction, and bone erosion. These features are very similar to the ones described by other authors in PGIA and also in human arthritis [13, 28]. This parallelism indicates that bovine PG can be further explored as another source of antigen to study arthritis. The efficacy of this bovine PG to induce murine arthritis is probably related, among other things, to the adopted adjuvant. As nicely described by Hanyecz et al. [23], the arthritogenicity of bovine PG was significantly incremented when it was combined with DDA. We also believe that the employment of BALB/c retired breeders contributed a lot to this achievement. According to Tarjanyi et al. [29], these old animals are very prone to arthritis development.

Immunization with this commercial product also induced significant production of IgG1 and IgG2b anti-bovine PG antibodies. Even though we were not able to assess a possible cross-reactivity of these antibodies with murine PG, we believe that it exists and is underlying the arthritogenicity of bovine PG to mice. This assumption is mainly based on structural and comparative biochemical studies and on arthritogenicity for mice. In this context, Walcz et al. [30] demonstrated that murine and bovine PG core protein share 72.5% homology. The arthritogenic potential of distinct PG sources was checked in mice. Interestingly, arthritogenicity or its absence was associated with the ability to induce or not, respectively, the production of cross-reactive antibodies [31].

An aspect that deserves further elucidation is the degree of glycosylation present in this commercial PG. It has been strongly emphasized that PG deglycosylation is fundamental to achieve arthritogenicity [13, 32]. However, we believe that this preparation is not devoid of polysaccharides. This hypothesis is based on references specified by the company that commercializes the product and also in information described by authors that utilized this product. According to Tham et al. [33], this commercial product contains 86% of chondroitin sulfate, 8% protein, 6% keratan sulfate, and less than 1% hyaluronic acid and it was able to enhance the survival of neural stem cells. In the central nervous system, chondroitin sulfate proteoglycan (CSPG) is the most prevalent PG and CS removal with chondroitinase reduces neural stem cells proliferation and neurogenesis. Also, according to Antonopoulos et al. [34], PG isolated by urea procedure is probably found in PG subunits instead of PG complexes form due to its gel chromatographic pattern. PG subunits could expose the G1 domain and the link protein, which are highly arthritogenic [27, 35]. Antonopoulos et al. [34] also demonstrated that urea did not cause PG degradation. It is possible to think that this organic compound could interfere in PG structure and protein solubility exposing some core protein epitopes and, therefore, become able to induce experimental arthritis.

Results from cytokine production by spleen cells* in vitro* stimulated with PG showed that arthritic animals, but not healthy ones, produced high levels of IL-2, TNF-*α*, IL-6, IFN-*γ*, and IL-17. Spleen cells from arthritic mice were already producing higher levels of IL-2 than the healthy ones. Also, addition of PG to the cultures determined a significant increase in the production of this cytokine in the arthritic group. As a very good correlation has been established between IL-2 level and T-cell proliferation index, in either up- [36] or downregulation [37], our results indicate the occurrence of a specific proliferative process in the spleen. The higher production of TNF-*α*, IL-6, IFN-*γ*, and IL-17 was expected and corroborates with their arthritogenic potential observed in humans [38] and in animal models [24]. The production of IL-6 and TNF-*α* is related to the immunopathogenesis and maintenance of RA. These cytokines are also responsible for hyperalgesia caused by mechanical stimulus in this disease. According to Schaible et al. [38], these proinflammatory cytokines act on nerve cells responsible for the nociceptive stimuli during joint movement. These authors showed that drugs which neutralize the action of TNF-*α* promoted reduction of pain and inflammation in rats with adjuvant-induced arthritis. A similar result was found concerning IL-6 neutralization. Thus, drugs whose action mechanisms are based on TNF-*α*, IL-6, and IL-1 neutralization have been extensively studied and some of them such as TNF-*α* and IL-6 are already used for the treatment of RA clinical symptoms [39].

Many studies have considered the balance between the production of IL-17 and IFN-*γ* as the key to understand the main immunopathogenic mechanisms involved in arthritis development. Considered a major proinflammatory cytokine in human arthritis and in most experimental models, IL-17 plays an important role in the establishment, maintenance, and progression of this disease [40–42]. Regarding this, studies have shown that the absence of IL-17 decreased the clinical symptoms of arthritis in different experimental models [43, 44]. The role of IL-17 in PGIA is not clearly evaluated yet. However, it has been suggested that in this case the IFN-*γ* is more important than IL-17 in the establishment of the disease. Doodes et al. [21], using knockout mice, showed that IFN-*γ* is essential for PGIA triggering in an IL-17 independent manner. The IFN-*γ* represents a paradox in autoimmune arthritis. Although its pathogenic effect is well described, recent studies showed a protective effect of this cytokine in arthritis. Alzabin and Williams [45] carefully reviewed the role of effector T cells in autoimmune arthritis. By analyzing the results of several experimental models, the authors demonstrated the protective role of IFN-*γ*. The administration of this cytokine that is, theoretically, proinflammatory, decreased clinical signals in different arthritis models. For example, genetically modified animals which were not able to produce IFN-*γ* presented an exacerbated collagen-induced arthritis [46]. However, it has been also reported that animals that did not produce this cytokine were less susceptible to PGIA [47].

The specific* in vitro* stimulation of spleen cells also triggered production of anti-inflammatory cytokines such as IL-5 and IL-10. Although RA is considered a disease characterized by predominant Th1 pattern, studies indicate that Th2 cytokines such as IL-4 and IL-10 also contribute to the immunopathogenesis of the disease and may also be related to the stage of disease development [48]. According to Gerli et al. [49], there is a high production of IL-4 and IL-10 by T cells from peripheral blood of patients in earlier stages of arthritis, but this production decreases significantly in later stages, contributing to disease progression and joint destruction in the chronic phase. Our results are, therefore, similar to the mixed Th1/Th2 pattern already shown in humans and in PGIA model [31]. An interesting aspect was observed in nonstimulated spleen cell cultures from arthritic animals when compared to control group. The arthritic group produced detectable levels of IFN-*γ*, TNF-*α*, IL-6, IL-17, IL-5, and IL-10 even in the absence of the specific stimulus. This spontaneous production, which might be more properly called endogenous production, could result from the intense immune response activation and cytokine secretion by effector cells that are significantly occurring.

Some interesting results mainly related to IL-17 were also detected in spleen cell cultures stimulated with ConA. In this case, we highlight the fact that the production of this cytokine after polyclonal stimulation was very similar to that induced by specific antigen stimulation. This finding is different from the ones usually obtained after polyclonal activation. The stimulation with mitogens is usually associated with induction of significantly higher cytokine production than the specific stimulus. However, recently, Doodes et al. [21] observed that the production of IL-17 and IFN-*γ* in response to specific stimulus is extremely high, reaching levels greater than 2000 pg/mL in the PGIA model. Similarly high levels of IL-17 were observed in human studies. Leipe et al. [50] evaluated the importance of IL-17 in autoimmune arthritis and found that purified T cells from the peripheral blood of patients, in the early stage of the disease, produced very high levels of this cytokine. Furthermore, analysis of the production of IL-17 by mononuclear cells from peripheral blood of healthy individuals, in response to different mitogens, revealed that the level of this cytokine in response to ConA did not exceed 500 pg/mL [51].

## 5. Conclusions

Our results indicate that this commercial bovine PG is highly arthritogenic for BALB/c retired breeder mice. In addition, the disease induced by this reagent presents clinical symptoms and histopathological features that are very similar to those found in other arthritis models and also the human corresponding pathology. Taken together, these results suggest that this bovine PG can be used as an alternative source in PGIA for the study of many aspects of RA, including the immunopathogenesis of the disease and also the development of new therapies.
